# Freshwater transitions and symbioses shaped the evolution and extant diversity of caridean shrimps

**DOI:** 10.1038/s42003-018-0018-6

**Published:** 2018-02-22

**Authors:** Katie E. Davis, Sammy De Grave, Cyrille Delmer, Matthew A. Wills

**Affiliations:** 1 Department of Biology, University of York, Wentworth Way, Heslington, York YO10 5DD UK; 2 Oxford University Museum of Natural History, Parks Road, Oxford, OX1 3PW UK; 3 Department of Biology and Biochemistry, University of Bath, Claverton Down, Bath, BA2 7AX UK

## Abstract

Understanding the processes that shaped the strikingly irregular distribution of species richness across the Tree of Life is a major research agenda. Changes in ecology may go some way to explain the often strongly asymmetrical fates of sister clades, and we test this in the caridean shrimps. First appearing in the Lower Jurassic, there are now ~3500 species worldwide. Carideans experienced several independent transitions to freshwater from marine habitats, while many of the marine species have also evolved a symbiotic lifestyle. Here we use diversification rate analyses to test whether these ecological traits promote or inhibit diversity within a phylogenetic framework. We demonstrate that speciation rates are more than twice as high in freshwater clades, whilst symbiotic ecologies are associated with lower speciation rates. These lower rates amongst symbiotic species are of concern given that symbioses often occur in some of the most diverse, delicately balanced and threatened marine ecosystems.

## Abstract (web summary)

Katie Davis et al. test the hypothesis that ecological traits are linked to diversification in caridean shrimps. They find that transitions from marine to freshwater habitats contributed to higher diversification rates, whereas symbiosis is associated with a slight decrease in diversification rates.

## Introduction

Developing a better understanding of the forces that shape global biodiversity patterns was identified as one of the 25 greatest challenges for Science in the 21st Century^1, 2^ and is an enduring problem in modern biology. There are, depending upon estimates, between 2 and 50 million extant species of animals (Metazoa)^3, 4^, all derived from a single common ancestral species that lived some 650 million years ago^5, 6^. Net rates of speciation therefore exceed rates of extinction, but the balance of these processes varies greatly, both between clades and throughout geological time^7, 8^. This asymmetry is often most marked in the differing fortunes of sister clades. For example, there are over a million living species of insects, but only 17 species of their sister group, the remipede crustaceans^9^. Changes in global temperature, habitat availability and ecological competition can all drive diversification and extinction on geological time scales^10–12^. In particular, transitions into new habitats and the evolution of key innovations (advantageous traits that allow the exploitation of new resources or habitats) may open up new ecological opportunities that may result in higher rates of speciation and therefore net diversification over time^13, 14^. Adaptive radiation theory predicts that following the colonisation of new habitats (or the acquisition of favourable characters), clades entering new adaptive space will undergo rapid lineage diversification as they fill this new, and previously unexploited, ecological space^15^.

The infraorder Caridea (commonly known as caridean shrimps) are a highly diverse group of decapod crustaceans, and second only to Brachyura (true crabs) in their species richness. More than 3,500 species had been described by 2011^16^, with a significant number of additions since then. Approximately 800 species live in freshwater and related continental waters (e.g., anchialine caves)^17^, amounting to nearly a quarter of global caridean diversity. Importantly, the transition from marine to freshwater habitats appears to have occurred independently within multiple branches (and at various hierarchical levels) of the caridean tree^18^. Transitions from marine to freshwater systems are well documented in both vertebrates and invertebrates and have been shown to increase speciation rates in amphipods^19^ and in fishes^20^. Moreover, several groups of marine carideans have independently evolved a symbiotic lifestyle, living in close association with poriferans, cnidarians, echinoderms, tunicates, molluscs and fishes, most noticeably the families Alpheidae and Palaemonidae^21^. The vast majority of these symbiotic carideans are associated with coral reef communities. Adapting to new ecological resources through symbioses may also increase rates of speciation^22, 23^.

Here we test the hypothesis that two key ecological traits for Caridea—namely broad habitat type (marine/freshwater) and symbioses—contributed to increased rates of diversification in the geological past via an adaptive radiation into new ecological space. We posit that this subsequently shaped the global patterns of caridean species diversity that we see today. To address this question, we first built a time-calibrated species-level supertree of Caridea. We coded the two traits as binary characters (marine/freshwater and free-living/symbiotic) and mapped them onto our new, inclusive phylogeny. We then used Ancestral State Reconstruction (ASR) to infer how many times each trait transitioned from the ancestral state. Clade-dependent diversification rates were then calculated to determine whether transitions into freshwater or the evolution of a symbiotic mode of life had any effect on net diversification rates through geological time.

We find that transitions into freshwater habitats resulted in net diversification rates more than double those found in marine clades. Conversely, the evolution of symbioses is associated with a small decrease in net diversification rates. The latter finding has implications for vulnerability assessments of symbiotic marine carideans, given that they are predominantly associated with coral reefs, which are amongst the most threatened ecosystems on Earth.

## Results

### Supertree construction

**Fig. 1:**
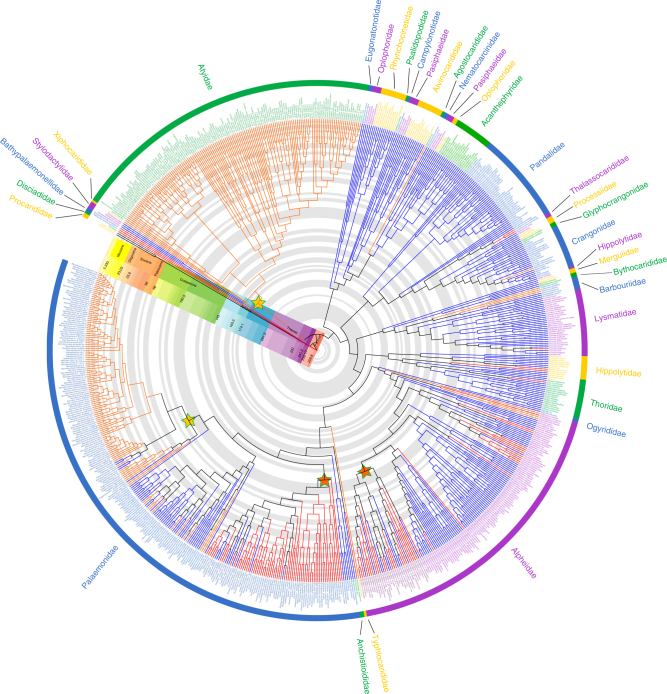
Phylogenetic tree of Caridea. Maximum Agreement Subtree (MAST) shown from MRP supertree analysis, scaled to geological time. Branch colouring was assigned as follows: blue = marine, free-living; red = marine, symbiotic; orange = freshwater, free-living. Stars indicate the node from which the clade rates for the diversification analyses were calculated (yellow = freshwater, orange = symbiotic). Geological time scale was added using the R package “strap”^27^

Using Matrix Representation with Parsimony (MRP)^24^, we inferred a phylogenetic supertree from 126 source trees taken from 66 papers published between 1984 and 2014. Although supertree methods, and MRP in particular, are not without their critics, this is still by far the most tractable approach for data sets of this size (1000 s taxa)^25^. Our resulting caridean supertree comprised 756 taxa (two Procarididea, the sister group to Caridea^26^, and 754 Caridea) and is the largest phylogeny of the group published to date (Fig. 1), being broadly consistent with recent discussions of their relationships^28–30^. All families are monophyletic with the exception of Oplophoridae, Pasiphaeidae and Hippolytidae. This taxonomic uncertainty is reflected in the source trees, and is not an artefact of the tree-building method. The non-monophyly of Pasiphaeidae has been suspected hitherto^31^, with a recent recalibration of the constituent genera^32^. Despite considerable progress towards resolving the problematic phylogeny of Hippolytidae *sensu lato*^30^, further studies found additional polyphyly^33^, consistent in generic scope with the present analysis. The division of the older concept of Oplophoridae into two families^34^ has remained controversial^35^, with one genus—*Systellaspis*—occupying an intermediate position between two families, as in the present analysis.

### Ancestral state reconstructions

For our ASR we collected trait data for all 756 species in the phylogeny. Freshwater taxa were defined as those permanently residing in freshwater or requiring freshwater to complete their lifecycle^17^. Species were considered to be “free-living” if, in general, they do not live on or inside a host animal^21^. Trait data on freshwater or marine habitats followed the IUCN Red List^36^ (based on De Grave et al.^17^). Anchialine and symbiotic trait states were collected from an exhaustive literature search. Our ASR analyses were carried out in PhyTools^37^ and indicated six independent transitions into a freshwater/anchialine habitat and a single reversal back to marine conditions within the genus *Palaemon*. Of these transitions, two resulted in speciose (>10 species) freshwater clades, namely the family Atyidae (approximately 470 spp.) and the genus *Macrobrachium* (Palaemonidae, approximately 240 spp.), while the remainder resulted in clades with fewer than ten species in each instance (Fig. 1). Symbioses evolved independently 13 times with a number of reversals. As with the habitat transitions, two of these instances of symbioses resulted in large speciose clades (Palaemonidae with an estimated 470 symbiotic spp. and Alpheidae with 300 spp.), while the others resulted in single isolated species or in clades with fewer than 10 species (Fig. 1). For ASR raw output see Supplementary Fig. 1.

### Diversification dynamics

**Fig. 2:**
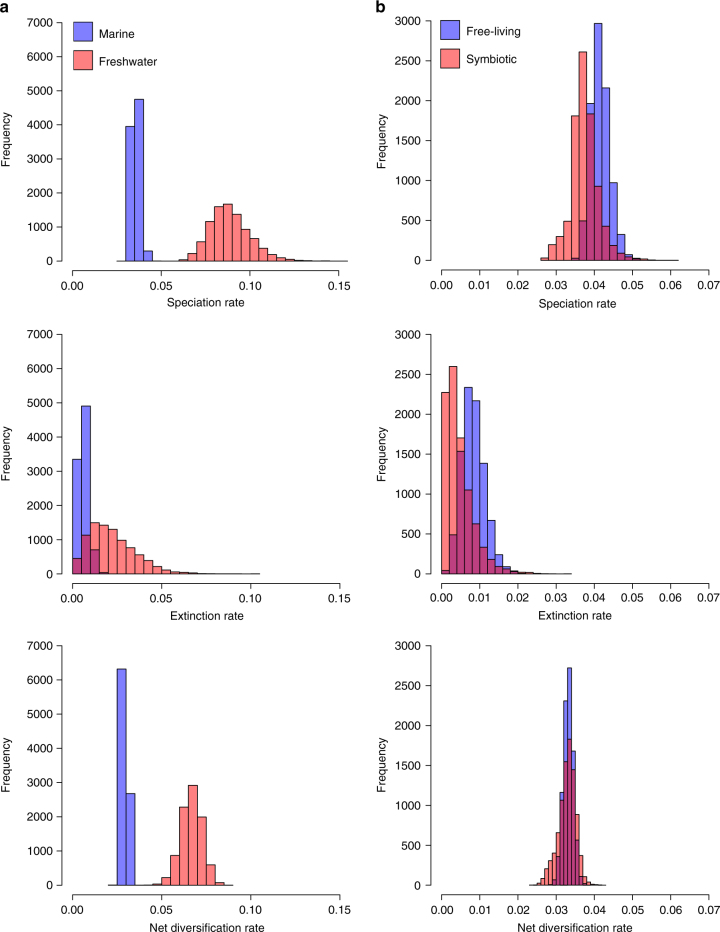
Clade-dependent diversification rate histograms. Frequency distributions from the BAMM analyses for speciation, extinction and net diversification rates for marine/freshwater clades (**a**) and free-living/symbiotic clades (**b**)

Using BAMM^14, 38^ to model speciation and extinction rates across the tree, we tested for significant associations between habitat or mode of life and clade-specific diversification rates (Fig. 2). We found that speciation rates were 2.5 times higher in freshwater clades than in their marine counterparts (marine: mean = 0.08881644, SD = 0.00219055; freshwater: mean = 0.03548732, SD = 0.01136907), while extinction rates in freshwater clades were more than 3.5 times higher than those in marine clades (marine: mean = 0.006113175, SD = 0.002677831; freshwater: mean = 0.02210151, SD = 0.0129926). Net diversification rates in freshwater clades were double those found in marine clades (marine: mean = 0.02937415; freshwater: mean = 0.06671493). Speciation rates were higher in free-living clades than in symbiotic clades by 1.1 and 1.8 times, respectively (free living: mean = 0.04152562, SD = 0.002447845; symbiotic: mean = 0.03748387, SD = 0.003546246), whereas extinction rates were higher by 1.8 times (free living: mean = 008334043, SD = 0.003015885; symbiotic: mean = 0.004622885, SD = 0.003668764). Net diversification rates in free-living clades were only slightly higher than in symbiotic clades (1.01 times higher) (free-living: mean = 0.03286099; symbiotic: mean = 0.03319158). As the rates were not normally distributed we used Wilcoxon rank and two-sample Kolmogorov–Smirnov tests to compare the posterior distribution of rate differences between each set of clades (marine vs. freshwater clades and free-living vs. symbiotic clades) and to assess significance. All the analyses were based on a sample size of 9000 sets of rates (10,000 minus burn-in), for each of speciation, extinction and net diversification rate, as calculated from the BAMM analyses. A two-sample Kolmogorov–Smirnov test was used to distinguish between the distributions of the 9000 mean rates for each clade pair while a Wilcoxon rank test compared the rates across all 9000 samples, but considered the differences between each clade pair (i.e., the comparison between freshwater/marine or free-living/symbiotic for a single simulation for which we have 9000 samples). Both tests showed that the difference in distributions between each trait pair was statistically significant (*P* < 2.2e–16 for speciation, extinction and net diversification rates for each trait pair). Overall, transitions into freshwater habitats appear to be associated with increased net rates of diversification, whereas transitions from a free-living to a symbiotic lifestyle are associated with reduced net rates of diversification.

If the traits investigated here—habitat and mode of life—are linked to changes in diversification rate, it might be expected that these rate shifts will show a correlation with changes in habitat and mode of life within clades as revealed by the ASR analyses. The BAMM analysis identified four significant rate shifts, all of which remain remarkably consistent in their timing and placement across the top nine credible shift configurations (see Supplementary Fig. 2 for the credible shift set). Two of these rate shifts are located within or at the base of the two major freshwater clades, a third is located at the base of a symbiotic clade, while the fourth (Pandalidae) shows no obvious link to either trait. Most of the significant rate shifts across the credible shift configurations were positive (more rapid diversification), with the exception of a shift associated with a symbiotic clade in two of the most likely configurations, for which rates decreased. Overall, the credible shift set provides stronger support for shifts associated with transitions to freshwater environments than for those associated with the evolution of symbioses.

## Discussion

In addition to vacant ecological space that the first colonists can exploit without competition^39, 40^, freshwater habitats also provide greater potential for allopatric speciation than marine environments. Although there are mechanisms that can promote allopatric speciation in marine settings—notably the fragmentation of shallow water environments by marine regressions^11^ and tectonic fragmentation^41^—freshwater habitats typically have much lower connectivity and far greater habitat isolation than found in the oceans. This lower connectivity in freshwater environments may also result in higher extinction rates^42^. Greater habitat fragmentation may limit the ability of a species to modify its distribution and geographical range in response to changes in climate^20^ and other environmental shifts. The greater geographical constraints of freshwater habitats are also associated with smaller niche width and a smaller geographic range, which are known to result in greater diversification rates in birds and mammals^43^. Although many freshwater shrimp are amphidromous (with larval dispersal via the sea) a significant proportion of Atyidae and *Macrobrachium* spp. have become landlocked with abbreviated larval development and therefore have extremely limited ranges; often a single lake or spring^17^. In birds and mammals, ecologically specialised species tend to be more threatened by extinction than generalists^44^; we demonstrate a similar pattern in Caridea, with almost a third of freshwater species being threatened with extinction^17^ (although a similarly detailed analysis of extinction risk in marine carideans is needed).

Our finding that rates of diversification are lowered in symbiotic clades seems counter intuitive. Many symbiotic shrimp have narrow host preferences and hence limited niche widths, which might be expected to result in higher diversification rates^45^. Symbioses and co-evolution have previously been shown to promote speciation in clownfish and sea anemones^22^, plants and insects^23^. However, simulations have shown that only certain types of co-evolutionary interactions promote speciation: in many cases it may even restrict diversification^45^. Symbiotic carideans require a good deal more systematic work, and much of their diversity remains to be described (e.g., within the sponge dwelling genus *Synalpheus*^46^), which may have influenced the analysis. Although many of these symbioses have long been thought to be commensal in nature, there is mounting evidence that a significant proportion of these assumed commensal species are actually situated on the parasitic spectrum^47^. For example, sponge-dwelling shrimp feed directly on their hosts, and although they actively prevent other individuals and species from colonising their sponge, this provides no discernible benefit to the host^47^. Parasitism can result in lower speciation rates if the behaviour of the parasite deleteriously affects the behaviour, appearance or sensory abilities of the host^48^. In strepsipteran insects, for example, the evolution of endoparasitism ultimately depressed speciation rates in the wake of an initial burst^49^.

We conjecture that this reduction in speciation rate may explain the frequent host switching seen within Palaemonidae^21^; the family with the highest percentage (60–80%) of symbiotic species^21^. Indeed, it appears that host-shift speciation could occur more readily than speciating further on a limited set of hosts^50^. Our finding of declining speciation rates in symbiotic species is particularly concerning. Such species are predominantly found within coral reef systems, which comprise some of the Earth’s most diverse, delicately balanced and threatened marine ecosystems. Unlike their freshwater counterparts, marine carideans have yet to have their extinction risk status assessed. Corals, however, which provide the host species to numerous symbiotic carideans are facing enormous threats from ongoing anthropogenic climate change, with a third of all species classified as ‘at risk’ according to the IUCN^36^. Future work should integrate macro-evolutionary and macro-ecological data through deep time with information on extinction risk for living species in order to reduce species losses due to climate change.

## Methods

### Source tree collection

Potential source trees were identified from online resources. The Web of Knowledge Science Citation Index^51^ was searched from 1980 to 2014 using the search terms: phylog*, taxonom*, systematic*, divers*, cryptic and clad* in conjunction with all scientific and common names for Caridea from infra-order to sub-family level. All papers mentioning or implying the existence of a tree in their title or abstract were examined. All source trees and selected meta-data were digitised in their published form using TreeView^52^ and the Supertree Toolkit (STK^53^). The latter is a fully integrated set of scripts designed to process trees and meta-data, and to output matrices for MRP^24^ supertree analysis or sets of trees for analysis using other supertree methods. Meta-data included bibliographic information, the types of characters used (e.g., molecular or morphological) and the methods used for tree inference. No corrections were made for synonyms or any other apparent errors or inconsistencies in the source trees prior to processing. All the source tree data were deposited in the Supertree Toolkit database^54^.

### Data processing

All source trees were curated and analysed in a consistent and repeatable manner in assembling the supertree^55, 56^. Once data collection and data entry were complete, we ensured that source trees met the following three criteria before inclusion in the analysis:

1. Only trees presented by their authors as explicit reconstructions of evolutionary relationships were included. We also excluded taxonomies, informal phylogenies, and any other trees not derived from an explicit matrix of clearly identified characters.
2. Only trees comprising clearly identified species, genera or higher taxa were included.
3. Only trees derived from the analysis of a novel, independent data set were included. This avoided pseudo-replication of the source trees and spurious levels of support for the resampled relationships.

Non-independent studies were defined as those that utilised identical matrices (i.e., the same taxa and characters), or where one matrix was a subset of the other. In the former case, the source trees based on ‘identical’ data trees were weighted in inverse proportion to their number. In the latter case, the less inclusive tree was removed from the data set.

Operational taxonomic units (OTUs) were standardised to reduce the inclusion of higher taxa, and to remove synonyms and vernacular names (which were standardised using the freely available online WoRMS database^57^). Where authors used higher taxa as proxies for particular exemplars, we substituted those higher taxa with the names of the exemplar genera or species. Where no exemplars were specified, higher taxa were removed from source trees by substituting those constituent taxa present in other source trees as a polytomy in the focal tree. This avoided artificial inflation of the taxon sample. Definitions for higher taxa were derived from WoRMS^57^.

Taxonomic overlap was checked once the nomenclature had been standardised. Each source tree required at least two taxa in common with at least one other source tree. Overlap within our data set was sufficient; therefore no source trees were removed and we were able to proceed to matrix creation without any further edits. See Supplementary Data 1 for the source trees as they were included in the analysis and Supplementary Data 2 for a reference list for all source trees. Source trees in their original form were deposited in the Supertree Toolkit website database^54^.

### Supertree construction

Using Matrix Representation with Parsimony (MRP)^24^, we inferred a phylogenetic supertree from 126 source trees taken from 66 papers published between 1984 and 2014. Source trees were encoded as a series of group inclusion characters using standard Baum and Ragan coding^24^, and automated within the STK software^53^. All taxa subtended by a given node in a source tree were scored as ‘1’, taxa not subtended from that node were scored as ‘0’, and taxa not present in that source tree were scored as ‘?’. Trees were rooted with a hypothetical, ‘all zero’ outgroup. The resulting MRP matrix was analysed using standard parsimony algorithms in TNT^58^. We used the ‘xmult = 10’ option, and ran 1000 replicates for the analysis, each using a different random starting point for the heuristic search. This improved exploratory coverage of the tree space, potentially avoiding local minima in the solutions. We computed a Maximum Agreement Subtree (MAST) using PAUP*^59^ to remove conflicting leaves, reducing the number from 1028 to 854. We identified a small number of rogue taxa (~5%) in the resulting tree (see Supplementary Data 3). One disadvantage of the MRP method is that it can potentially lead to the creation of spurious clades and relationships that are not present in any of the source trees (novel clades)^11, 56, 60^. We refer to these misplaced taxa within novel clades as ‘rogue taxa’. Rogue taxa are usually a result of either poorly constrained or poorly represented taxa within in the source trees. Importantly, however, this problem is not limited to supertree methods. Studies have shown that identifying and removing rogue taxa a priori can create its own problems, as rogue taxa have the potential to phylogenetically constrain the positions of other taxa in the analysis. Hence a priori removal often simply creates new rogue taxa. It is important that these novel clades are not interpreted as biologically meaningful and are, instead, removed from the phylogeny before undertaking further analysis^61^. We therefore provide a full list of removed taxa in Supplementary Data 3.

### Phylogeny time-calibration

Supertrees derived from parsimony analyses do not contain branch lengths that can be used to infer dates of relative splits, rates of evolution or rates of diversification. Rather, branch lengths in MRP supertrees reflect a parsimonious resolution of all the inferences of clade membership across the set of source trees; inferences that are potentially (and often) mutually incompatible. In order to time-scale we therefore used a combination of direct fossil calibration supplemented by inferences from molecular analyses. Six nodes were calibrated using fossil first occurrence data^62^. Fossils selected for calibration were those that conclusively showed the characteristics of the family concerned, and assigned to clades using the decapod genus list classification^63^. As there are so few reliable caridean fossils, we obtained additional calibration points from published molecular phylogenetic analyses^26, 64^ (see Supplementary Table 1 for node numbers and calibration dates and Supplementary Figure 3 for the tree with the calibrated nodes labelled as in the CSV file). The R package ‘paleotree’^65^ was used to scale the tree and extrapolate dates to the remaining nodes. To extend node calibration to the whole tree, we used the ‘equal’ method, with minimum branch lengths set to 0.1 Myr. See Supplementary Data 4 for the time-calibrated supertree in Nexus format.

### Ancestral state reconstruction

We used ASR to infer when, and how often, caridean shrimps transitioned between marine and freshwater habitats and also to infer the origin(s) of symbiotic relationships. We applied stochastic character mapping to the time-calibrated supertree, implemented using the ‘make.simmap’ in PhyTools^37^. The variables for habitat (freshwater/anchialine or marine) and symbiotic (yes or no) were both discrete and two-state, and were optimised using equal-rates models. See Supplementary Tables 2 and 3 for species trait lists and Supplementary Data 5 for the R code.

### Diversification rates

Diversification rates were assessed using BAMM^14, 38^, which implements an MCMC approach to calculate diversification rates and significant rate shifts. Four chains were executed, each running a total of 30 million generations, with a minimum clade size of five taxa used to aid convergence. Ten thousand of the resulting trees were stored, with 1000 discarded as ‘burn-in’, leaving 9000 samples for subsequent analysis. The analysis also accounted for non-complete coverage of taxa in the tree by specifying a clade-dependent sampling bias factor derived from taxonomy^63^. For full details of our sampling regime and BAMM implementation, see Supplementary Data 6 and 7.

### Clade-dependent diversification rate correlations

We explored the effects of two different ecological traits; habitat and mode of life, on diversification rates of lineages through time. All taxa were designated as either marine or freshwater/anchialine and as symbiotic or free-living. We then used BAMMtools^38^ to extract diversification rates from the posterior distributions of rates from our BAMM analyses. We obtained mean rates of speciation, extinction and net diversification for all freshwater and marine clades, and all symbiotic and non-symbiotic clades. For these analyses we only considered clades that contained >10 taxa. The rates compared were taken from the BAMM analyses, which consist of 10,000 samples generated from 30 million generations. Ten percent are then discarded for burn-in, leaving a sample size of 9000. The resulting mean rates were not normally distributed. We therefore used two statistical tests; the Wilcoxon signed rank test to determine whether the median mean rates were different; and the two-sample Kolmogorov–Smirnov test to determine whether the distributions of mean rates (both central tendency and shape) were different.

### Data availability

The authors declare that all data supporting the findings of this study are available within the article and its supplementary information files.

## Electronic supplementary material

Supplementary Information

Description of Additional Supplementary Files

Supplementary Data 1

Supplementary Data 2

Supplementary Data 3

Supplementary Data 4

Supplementary Data 5

Supplementary Data 6

Supplementary Data 7
