# Supplementary material for: Freshwater transitions and symbioses shaped the evolution and extant diversity of caridean shrimps
Source: Commun Biol. 2018 Feb 22;1:16. doi: 10.1038/s42003-018-0018-6 (PMC6123698; doi:10.1038/s42003-018-0018-6)
Supplement: Supplementary file 1 — Supplementary Information [file 42003_2018_18_MOESM1_ESM.pdf]

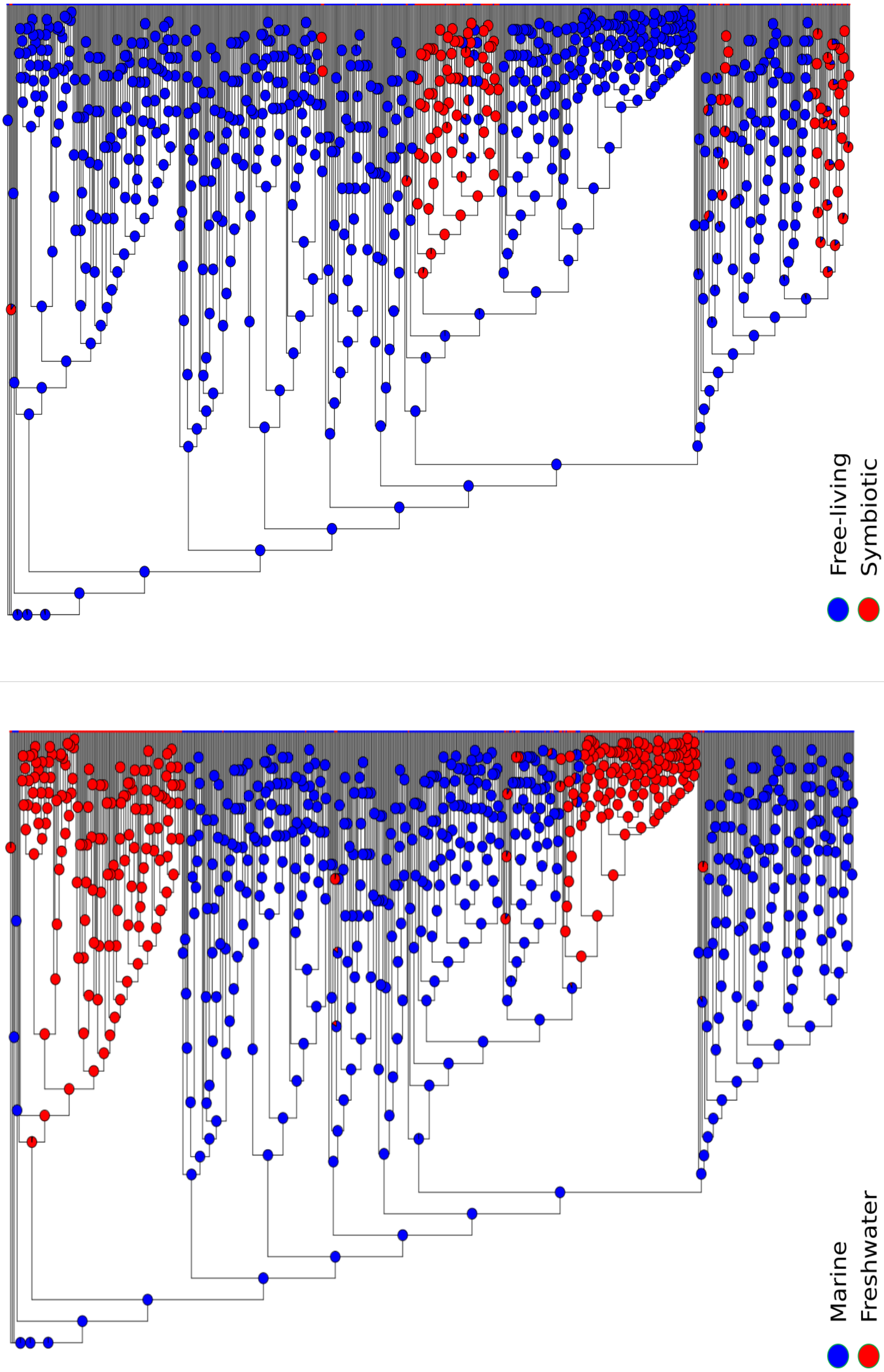

Supplementary Figure 1: Ancestral State Reconstructions (ASR) for both habitat and mode of life. Raw output from Ancestral State Reconstruction as computed in PhyTools[1]. Left: marine (blue) vs freshwater (red); right: free-living (blue) vs symbiotic (red).

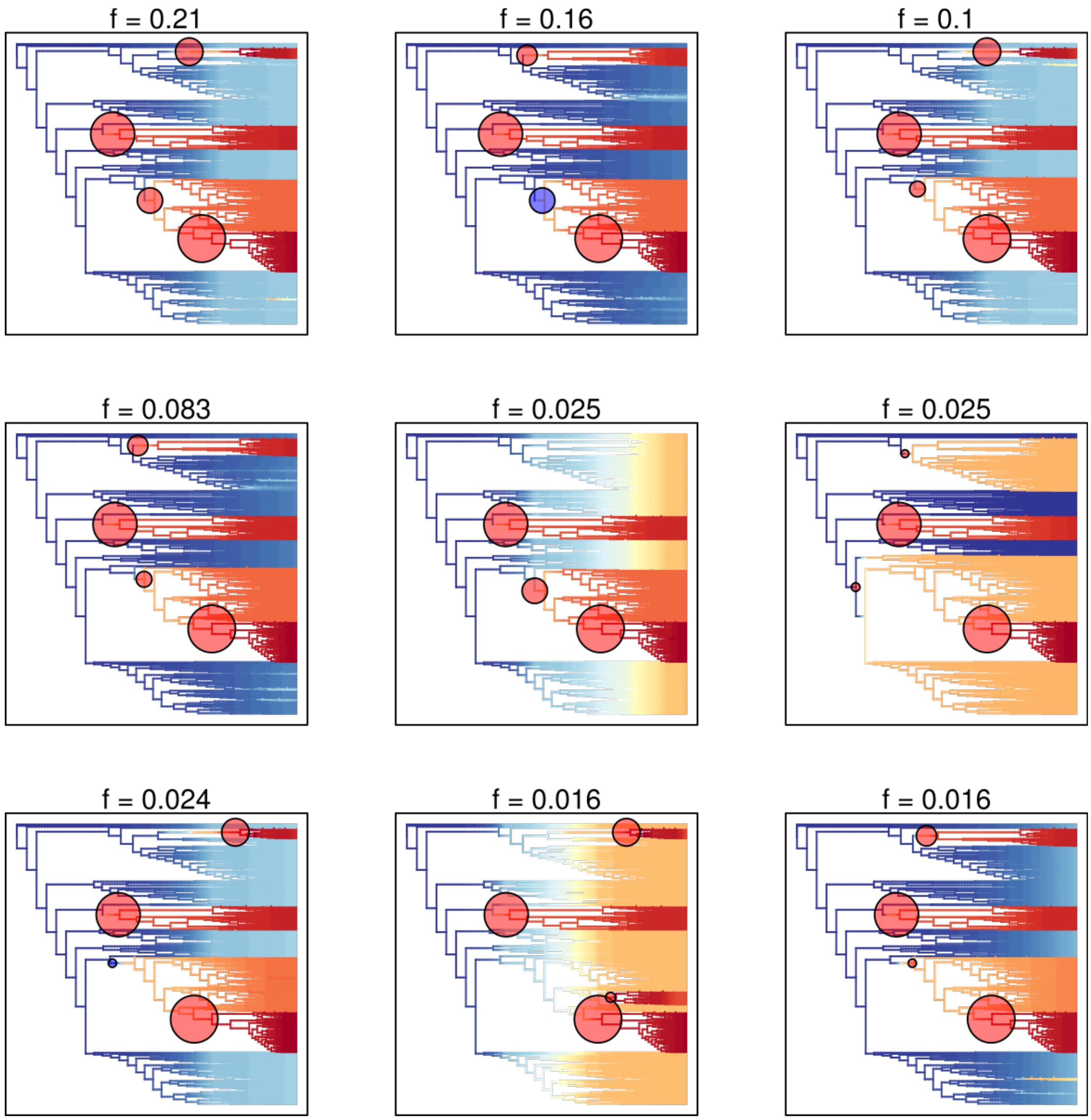

Supplementary Figure 2: Set of nine most probable shift configurations (credible shift set). These nine configurations as computed in BAMM[2,3] are remarkably stable, with little change in the number or position of the diversification rate shifts.

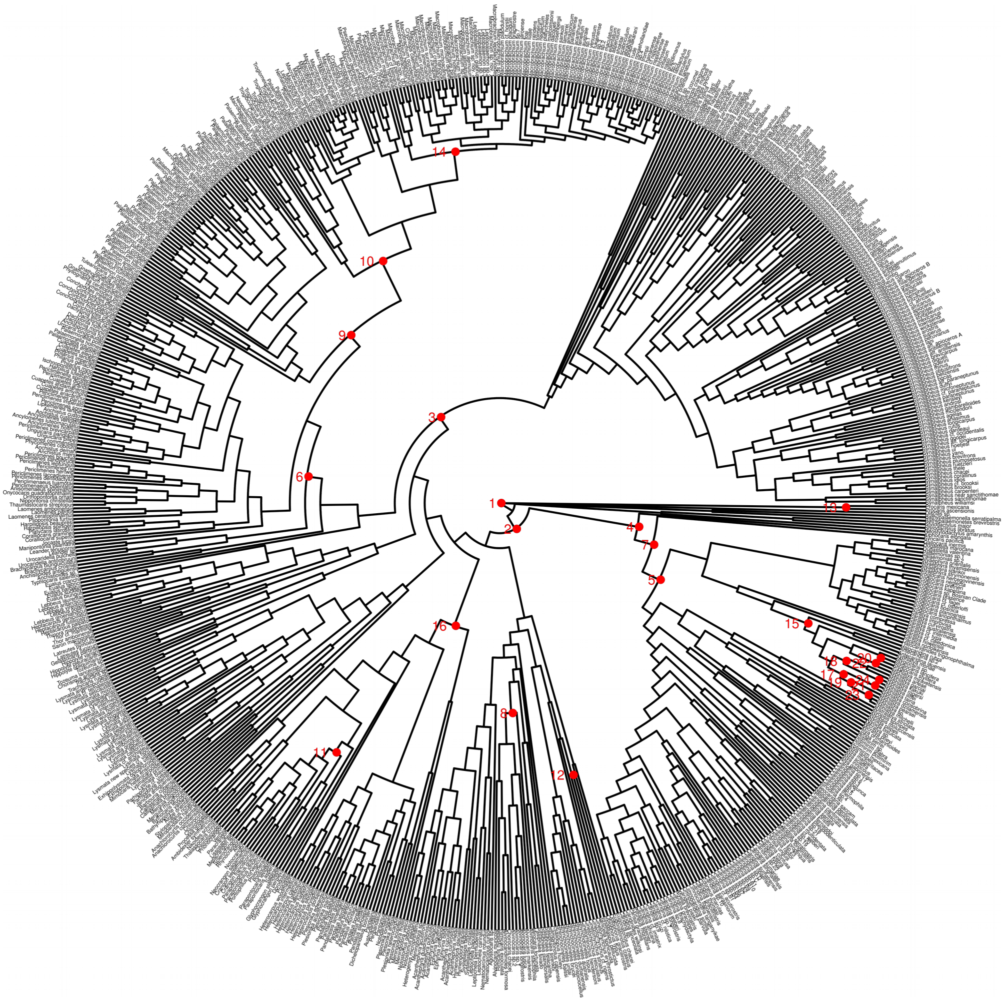

Supplementary Figure 3: Labelled nodes used for fossil and molecular calibrations points for time-scaling the supertree.

Supplementary Table 1

Node number	Fossil	Molecular tree	FAD	LAD
1	N/A	Bracken *et al*.,. 2010.	262.63	262.63
2	N/A	Bracken *et al*.,. 2010.	244.15	244.15
3	N/A	Bracken *et al*.,. 2010.	198	198
4	N/A	Bracken *et al*.,. 2010.	176.4	176.4
5	N/A	Bracken *et al*.,. 2010.	153.6	153.6
6	*Schmelingia wulfi*	N/A	152.1	145
7	*Harthofia bergeri*	N/A	152.1	145
8	*Harthofia blumenbergi; Harthofia polzi*	N/A	152.1	145
9	N/A	Bracken *et al*.,. 2010.	123.82	123.82
10	N/A	Bracken *et al*.,. 2010.	96.64	96.64
11	*Odontochelion cretaceum*	N/A	86.3	83.6
12	*Oplophorus marcki; Oplophorus syriacus*	N/A	89.3	65.5
13	N/A	Bracken *et al*.,. 2010.	49.9	49.9
14	N/A	Bracken *et al*.,. 2010.	44.27	44.27
15	N/A	Botello *et al*., 2013.	59.24	28.17
16	*Crangon miocenicus*	N/A	23.03	15.97
17	N/A	Botello *et al*., 2013.	26.54	14.24
18	N/A	Botello *et al*., 2013.	28.46	13.97
19	N/A	Botello *et al*., 2013.	20.22	9.7
20	N/A	Botello *et al*., 2013.	9.75	7
21	N/A	Botello *et al*., 2013.	5.99	5.05
22	N/A	Botello *et al*., 2013.	11.34	4.18
23	N/A	Botello *et al*., 2013.	6.9	2.7
24	N/A	Botello *et al*., 2013.	5.26	1.88

References:

Bracken, H. D., De Grave, S., Toon, A., Felder, D. L. & Crandall, K. A. Phylogenetic position, systematic status, and divergence time of the Procarididea (Crustacea: Decapoda). Zoologica Scripta 39, 198–212 (2010).

Botello, A., Iliffe, T. M., Alvarez, F., Juan, C., Pons, J. & Jaume, D. Historical biogeography and phylogeny of *Typhlatya* cave shrimps (Decapoda: Atyidae) based on mitochondrial and nuclear data. Journal of Biogeography 40, 594–607 (2013).

Supplementary Table 2

Taxon	Freshwater/ anchialine	Marine	Taxon	Freshwater/ anchialine	Marine
Acanthephyra_acutifrons	0	1	Anchistioides_antiguensis	0	1
Acanthephyra_cucullata	0	1	Anchistioides_sp.1	0	1
Acanthephyra_curtirostris	0	1	Anchistus_custoides	0	1
Acanthephyra_media	0	1	Anchistus_custos	0	1
Acanthephyra_pelagica	0	1	Anchistus_demani	0	1
Acanthephyra_purpurea	0	1	Anchistus_miersi	0	1
Acanthephyra_quadrispinosa	0	1	Ancylomenes_holthuisi	0	1
Agostocaris_sp.	1	0	Ancylomenes_luteomaculatus	0	1
Alope_orientalis	0	1	Ancylomenes_venustus	0	1
Alpheopsis_aequalis	0	1	Anisomenaeus_spinimanus	0	1
Alpheopsis_trispinosa	0	1	Antecaridina_lauensis	1	0
Alpheus_agilis	0	1	Antecaridina_sp.2	1	0
Alpheus_amblyonyx	0	1	Arete_indicus	0	1
Alpheus_antepaenultimus	0	1	Aretopsis_amabilis	0	1
Alpheus_bouvieri	0	1	Argis	0	1
Alpheus_chacei	0	1	Athanas_dimorphus	0	1
Alpheus_colombiensis	0	1	Athanas_nitescens	0	1
Alpheus_cristulifrons	0	1	Athanas_squillophilus	0	1
Alpheus_cylindricus	0	1	Athanopsis_australis	0	1
Alpheus_dentipes	0	1	Athanopsis_brevirostris	0	1
Alpheus_edwardsii	0	1	Athanopsis_dentipes	0	1
Alpheus_estuariensis	0	1	Athanopsis_platyrhynchus	0	1
Alpheus_floridanus	0	1	Athanopsis_rubricinctuta	0	1
Alpheus_formosus	0	1	Atya_gabonensis	1	0
Alpheus_galapagensis	0	1	Atya_innocous	1	0
Alpheus_hebes	0	1	Atya_lanipes	1	0
Alpheus_javieri	0	1	Atya_margaritacea	1	0
Alpheus_latus	0	1	Atya_ortmannioides	1	0
Alpheus_macrocheles	0	1	Atya_scabra	1	0
Alpheus_malleator	0	1	Atyaephyra_orientalis	1	0
Alpheus_normanni	0	1	Atyaephyra_stankoi	1	0
Alpheus_nuttingi	0	1	Atyaephyra_strymonensis	1	0
Alpheus_panamensis	0	1	Atyaephyra_thyamisensis	1	0
Alpheus_paracrinitus	0	1	Atyella_brevirostris	1	0
Alpheus_peasei	0	1	Atyoida_bisulcata	1	0
Alpheus_rapacida	0	1	Atyoida_pilipes	1	0
Alpheus_rostratus	0	1	Atyopsis_moluccensis	1	0
Alpheus_saxidomus	0	1	Atyopsis_spinipes	1	0
Alpheus_schmitti	0	1	Australatya_striolata	1	0
Alpheus_simus	0	1	Austropandalus	0	1
Alpheus_sulcatus	0	1	Automate_dolichognatha	0	1
Alpheus_tenuis	0	1	Automate_evermanni	0	1
Alpheus_thomasi	0	1	Automate_hayashii	0	1
Alpheus_umbo	0	1	Bannereus_anomalus	0	1
Alpheus_viridari	0	1	Barbouria_cubensis	1	0
Alpheus_websteri	0	1	Batella_parvimanus	0	1
Alvinocaridinides_formosa	0	1	Bathyhippolyte_yaldwyni	0	1
Alvinocaris_longirostris	0	1	Bathypalaemonella_serratipalma	0	1
Alvinocaris_lusca	0	1	Bathypalaemonetes_brevirostris	0	1
Alvinocaris_markensis	0	1	Bermudacaris_harti	0	1
Alvinocaris_muricola	0	1	Brachycarpus_biunguiculatus	0	1
Alvinocaris_sp.1	0	1	Brachycarpus_crosnieri	0	1
Alvinocaris_sp.2	0	1	Bythocaris_leucopis	0	1
Alvinocaris_stactophila	0	1	Calliasmata_nohochi	1	0
Ambidexter_symmetricus	0	1	Calliasmata_pholidota	1	0
Anachlorocurtis_commensalis	0	1	Campylonotus_capensis	0	1
Anachlorocurtis_occidentalis	0	1	Caridella_paski	1	0
Anchiopontonia_hurii	0	1	Caridina_africana	1	0

Supplementary Table 2

Taxon	Freshwater/ anchialine	Marine	Taxon	Freshwater/ anchialine	Marine
Caridina_brachydactyla	1	0	Caridinides_wilkinsi	1	0
Caridina_cantonensis	1	0	Chlorocurtis	0	1
Caridina_cf._acutirostris	1	0	Chlorotocella	0	1
Caridina_cf._imitatrix	1	0	Chlorotocoides	0	1
Caridina_cf._nilotica	1	0	Chorismus_antarcticus	0	1
Caridina_cf._weberi	1	0	Chorocaris_chacei	0	1
Caridina_confusa	1	0	Cineotorhynchus_cf._rigens	0	1
Caridina_cruzi	1	0	Cinetorhynchus_erythrostictus	0	1
Caridina_fernandoi	1	0	Cinetorhynchus_hendersoni	0	1
Caridina_gracilipes	1	0	Cinetorhynchus_reticulatus	0	1
Caridina_gracilirostris	1	0	Cinetorhynchus_striatus	0	1
Caridina_lanceolata	1	0	Conchodytes_biunguiculatus	0	1
Caridina_longirostris	1	0	Conchodytes_chadi	0	1
Caridina_multidentata	1	0	Conchodytes_meleagrinae	0	1
Caridina_opaensis	1	0	Conchodytes_monodactylus	0	1
Caridina_pristis	1	0	Conchodytes_placunae	0	1
Caridina_propinqua	1	0	Conchodytes_pteriae	0	1
Caridina_pseudodenticulata	1	0	Coralliocaris_graminea	0	1
Caridina_sarasinorum	1	0	Coralliocaris_superba	0	1
Caridina_serrata	1	0	Coronalpheus_natator	0	1
Caridina_serratirostris	1	0	Crangon_franciscorum	0	1
Caridina_sp.	1	0	Crangon_septemspinosa	0	1
Caridina_sp.1	1	0	Creaseria_morleyi	0	1
Caridina_sp.17	1	0	Cryphiops_brasiliensis	1	0
Caridina_sp.18	1	0	Cryphiops_caementarius	1	0
Caridina_sp.19	1	0	Cryphiops_luscus	1	0
Caridina_sp.2	1	0	Cuapetes_amymone	0	1
Caridina_sp.2	1	0	Cuapetes_anacanthus	0	1
Caridina_sp.21	1	0	Cuapetes_andamanensis	0	1
Caridina_sp.22	1	0	Cuapetes_elegans	0	1
Caridina_sp.23	1	0	Cuapetes_ensifrons	0	1
Caridina_sp.3	1	0	Cuapetes_grandis	0	1
Caridina_sp.4	1	0	Cuapetes_tenuipes	0	1
Caridina_sp.5	1	0	Dactylonia_ascidicola	0	1
Caridina_sp.6	1	0	Dactylonia_holthuisi	0	1
Caridina_sp.7	1	0	Dactylonia_monnioti	0	1
Caridina_sp.8	1	0	Dactylonia_okai	0	1
Caridina_sp.9	1	0	Deioneus_sandizelli	0	1
Caridina_sp.A1	1	0	Dichelopandalus_bonnieri	0	1
Caridina_sp.A2	1	0	Discias_sp.	0	1
Caridina_sp.B1	1	0	Dorodotes	0	1
Caridina_sp.B1	1	0	Dugastella_marocana	1	0
Caridina_sp.B4	1	0	Dugastella_valentina	1	0
Caridina_sp.B6	1	0	Edoneus_marulas	1	0
Caridina_sp.B7	1	0	Ephyrina_bifida	0	1
Caridina_sp.B9	1	0	Ephyrina_figueirai	0	1
Caridina_sp.C	1	0	Ephyrina_ombango	0	1
Caridina_sp.D	1	0	Eualus_barbatus	0	1
Caridina_spelunca	1	0	Eualus_biunguis	0	1
Caridina_steineri	1	0	Eualus_cranchii	0	1
Caridina_sumatrensis	1	0	Eualus_fabricii	0	1
Caridina_thermophila	1	0	Eualus_suckleyi	0	1
Caridina_thomasi	1	0	Eugonatonotus_chacei	0	1
Caridina_togoensis	1	0	Eugonatonotus_crassus	0	1
Caridina_trifasciata	1	0	Exhippolysmata_ensirostris	0	1
Caridina_typus	1	0	Exhippolysmata_oplophoroides	0	1
Caridina_weberi	1	0	Gallocaris_inermis	1	0
Caridina_zebra	1	0	Gelastocaris_paronae	0	1

Supplementary Table 2

Taxon	Freshwater/ anchialine	Marine	Taxon	Freshwater/ anchialine	Marine
Glyphocrangon_perplexa	0	1	Limnocaridina_similis	1	0
Glyphocrangon_stenolepis	0	1	Limnocaridina_tanganyikae	1	0
Gnathophylloides_mineri	0	1	Lipkemenes_lanipes	0	1
Gnathophyllum_americanum	0	1	Lissosabinea	0	1
Halocaridina_rubra	1	0	Lysmata_amboinensis	0	1
Halocaridinides_trigonophthalma	1	0	Lysmata_ankeri	0	1
Harpiliopsis_beaupresii	0	1	Lysmata_argentopunctata	0	1
Harpiliopsis_spinigera	0	1	Lysmata_bahia	0	1
Harpilius_lutescens	0	1	Lysmata_boggessi	0	1
Heptacarpus_futilirostris	0	1	Lysmata_californica	0	1
Heptacarpus_geniculatus	0	1	Lysmata_cf._anchisteus	0	1
Heptacarpus_palpator	0	1	Lysmata_cf._intermedia	0	1
Heterocarpus_amacula	0	1	Lysmata_cf._ternatensis	0	1
Heterocarpus_calmani	0	1	Lysmata_debelius	0	1
Heterocarpus_dorsalis	0	1	Lysmata_galapagensis	0	1
Heterocarpus_gibbosus	0	1	Lysmata_grabhami	0	1
Heterocarpus_hayashii	0	1	Lysmata_gracilirostris	0	1
Heterocarpus_intermedius	0	1	Lysmata_hochi	0	1
Heterocarpus_laevigatus	0	1	Lysmata_holthuisi	0	1
Heterocarpus_parvispina	0	1	Lysmata_intermedia	0	1
Heterocarpus_sibogae	0	1	Lysmata_moorei	0	1
Heterocarpus_woodmasoni	0	1	Lysmata_nayaritensis	0	1
Heterogenys_microphthalma	0	1	Lysmata_new_species_cf._vittata	0	1
Hippolyte_obliquimanus	0	1	Lysmata_nilita	0	1
Hippolyte_pleuracantha	0	1	Lysmata_olavoi	0	1
Hippolyte_varians	0	1	Lysmata_pederseni	0	1
Hymenocera_picta	0	1	Lysmata_rafa	0	1
Hymenodora_glacialis	0	1	Lysmata_seticaudata	0	1
Hymenodora_gracilis	0	1	Lysmata_udoi	0	1
Ischnopontonia_lophos	0	1	Lysmata_vittata	0	1
Izucaris_masudai	0	1	Lysmata_wurdemanni	0	1
Janicea_antiguensis	0	1	Lysmatella_prima	0	1
Janicella_spinicauda	0	1	Macrobrachium_abrahami	1	0
Jocaste_lucina	0	1	Macrobrachium_acanthurus	1	0
Jonga_serrei	1	0	Macrobrachium_aemulum	1	0
Lancaris_kumariae	1	0	Macrobrachium_amazonicum	1	0
Lancaris_singhalensis	1	0	Macrobrachium_americanum	1	0
Laomenes_amboinensis	0	1	Macrobrachium_aracamuni	1	0
Laomenes_ceratophthalmus	0	1	Macrobrachium_asperulum	1	0
Laomenes_pardus	0	1	Macrobrachium_australe	1	0
Latreutes_fucorum	0	1	Macrobrachium_birai	1	0
Latreutes_pymoeus	0	1	Macrobrachium_borellii	1	0
Leander_sp.	0	1	Macrobrachium_brasiliense	1	0
Leander_tenuicornis	0	1	Macrobrachium_carcinus	1	0
Lebbeus_antarcticus	0	1	Macrobrachium_cf_nipponense	1	0
Lebbeus_groenlandicus	0	1	Macrobrachium_cf_pilimanus	1	0
Lebbeus_laurentae	0	1	Macrobrachium_cf._horstii	1	0
Lebbeus_polaris	0	1	Macrobrachium_cortezi	1	0
Lebbeus_virentova	0	1	Macrobrachium_crenulatum	1	0
Leptocarpus_potamiscus	0	1	Macrobrachium_dacqueti	1	0
Leptochela_bermudensis	0	1	Macrobrachium_depressimanum	1	0
Leptochela_carinata	0	1	Macrobrachium_digueti	1	0
Leptochela_papulata	0	1	Macrobrachium_dux	1	0
Leptopalaemon_glabrus	1	0	Macrobrachium_edentatum	1	0
Leptopalaemon_sp.1	1	0	Macrobrachium_esculentum	1	0
Leptopalaemon_sp.2	1	0	Macrobrachium_ferreirai	1	0
Leptopalaemon_sp.3	1	0	Macrobrachium_foai	1	0
Limnocaridina_parvula	1	0	Macrobrachium_formosense	1	0

Supplementary Table 2

Taxon	Freshwater/ anchialine	Marine	Taxon	Freshwater/ anchialine	Marine
Macrobrachium_fukienense	1	0	Macrobrachium_sp.6	1	0
Macrobrachium_gracilirostre	1	0	Macrobrachium_sp.7	1	0
Macrobrachium_grandimanus	1	0	Macrobrachium_sp.8	1	0
Macrobrachium_hainanense	1	0	Macrobrachium_sp.nov._GUI	1	0
Macrobrachium_hancocki	1	0	Macrobrachium_surinamicum	1	0
Macrobrachium_heterochirus	1	0	Macrobrachium_tenellum	1	0
Macrobrachium_holthuisi	1	0	Macrobrachium_tolmerum	1	0
Macrobrachium_iheringi	1	0	Macrobrachium_trompii	1	0
Macrobrachium_inflatum	1	0	Macrobrachium_venustum	1	0
Macrobrachium_inpa	1	0	Macrobrachium_villalobosi	1	0
Macrobrachium_jaroense	1	0	Macrobrachium_yui	1	0
Macrobrachium_lamarrei	1	0	Macrobrachium_zariquieyi	1	0
Macrobrachium_lanatum	1	0	Manipontonia_psamathe	0	1
Macrobrachium_lar	1	0	Marosina_longirostris	1	0
Macrobrachium_latidactylus	1	0	Meningodora_mollis	0	1
Macrobrachium_latimanus	1	0	Meningodora_sp.	0	1
Macrobrachium_lepidactyloides	1	0	Meningodora_vesca	0	1
Macrobrachium_lujae	1	0	Merguia_oligodon	0	1
Macrobrachium_maculatum	1	0	Merguia_rhizophorae	0	1
Macrobrachium_malayanum	1	0	Metabetaeus_minutus	0	1
Macrobrachium_malcolmsonii	1	0	Metabetaeus_sp.	0	1
Macrobrachium_michoacanus	1	0	Metacrangon_sp.	0	1
Macrobrachium_naso	1	0	Metalpheus_rostratipes	0	1
Macrobrachium_nattereri	1	0	Micratya_poeyi	1	0
Macrobrachium_neglectum	1	0	Micratya_sp.	1	0
Macrobrachium_nepalense	1	0	Mimocaris_heterocarpoides	0	1
Macrobrachium_niphanae	1	0	Mirocaris_fortunata	0	1
Macrobrachium_nipponense	1	0	Miropandalus_hardingi	0	1
Macrobrachium_novaehollandiae	1	0	Nematocarcinus_cursor	0	1
Macrobrachium_occidentale	1	0	Nematocarcinus_rotundus	0	1
Macrobrachium_ohione	1	0	Nematopalaemon_schmitti	0	1
Macrobrachium_panamense	1	0	Nennalpheus_inarticulatus	0	1
Macrobrachium_pectinatum	1	0	Nennalpheus_sibogae	0	1
Macrobrachium_petronioi	1	0	Neocaridina_denticulata	1	0
Macrobrachium_pilimanus	1	0	Neocaridina_palmata	1	0
Macrobrachium_placidulum	1	0	Neocaridina_saccam	1	0
Macrobrachium_placidum	1	0	Neocrangon_sagamiensis	1	0
Macrobrachium_platycheles	1	0	Neopalaemon_nahuatlus	1	0
Macrobrachium_potiuna	1	0	Neostylodactylus_amarynthis	0	1
Macrobrachium_prabhakarani	1	0	Nikoides_schmitti	0	1
Macrobrachium_pumilum	1	0	Nippontonia_christellae	0	1
Macrobrachium_qilianensis	1	0	Notocrangon	0	1
Macrobrachium_quelchi	1	0	Notopandalus	0	1
Macrobrachium_raridens	1	0	Notostomus_elegans	0	1
Macrobrachium_reyesi	1	0	Notostomus_gibbosus	0	1
Macrobrachium_rodriguezi	1	0	Ogyrides_orientalis	0	1
Macrobrachium_rosenbergii	1	0	Ogyrides_sp.	0	1
Macrobrachium_rude	1	0	Onycocaris_quadratophthalma	0	1
Macrobrachium_sankolli	1	0	Oplophorus_gracilirostris	0	1
Macrobrachium_scabriculum	1	0	Oplophorus_typus	0	1
Macrobrachium_shokitai	1	0	Orthopontonia_ornata	0	1
Macrobrachium_sp._nov._1	1	0	Palaemon_adspersus	0	1
Macrobrachium_sp._nov._2	1	0	Palaemon_africanus	0	1
Macrobrachium_sp.1	1	0	Palaemon_annandalei	0	1
Macrobrachium_sp.2	1	0	Palaemon_antennarius	0	1
Macrobrachium_sp.3	1	0	Palaemon_antrorum	1	0
Macrobrachium_sp.4	1	0	Palaemon_argentinus	0	1
Macrobrachium_sp.5	1	0	Palaemon_atrinubes	1	0

Supplementary Table 2

Taxon	Freshwater/ anchialine	Marine	Taxon	Freshwater/ anchialine	Marine
Palaemon_carinicauda	0	1	Pandalus_danae	0	1
Palaemon_carteri		0	Pandalus_goniurus	0	1
Palaemon_concinnus	0	1	Pandalus_hypsinotus	0	1
Palaemon_cummingi	1	0	Pandalus_jordani	0	1
Palaemon_debilis	0	1	Pandalus_latirostris	0	1
Palaemon_elegans	0	1	Pandalus_montagui	0	1
Palaemon_floridanus	0	1	Pandalus_prensor	0	1
Palaemon_gladiator	0	1	Pandalus_stenolepis	0	1
Palaemon_gracilis	0	1	Pantomus	0	1
Palaemon_gravieri	0	1	Parabetaeus_culliereti	0	1
Palaemon_guangdongensis	0	1	Parabetaeus_euryone	0	1
Palaemon_hancocki	0	1	Paracrangon	0	1
Palaemon_hiltoni	0	1	Parapontocaris_aspera	0	1
Palaemon_holthuisi	0	1	Parapontocaris_levigata	0	1
Palaemon_intermedius	0	1	Parapontophilus_gracilis	0	1
Palaemon_ivonicus	0	1	Parapontophilus_junceus	0	1
Palaemon_litoreus	0	1	Paratya_australiensis	1	0
Palaemon_longirostris	0	1	Paratya_cf._caledonica	1	0
Palaemon_macrodactylus	0	1	Paratya_cf._intermedia	1	0
Palaemon_maculatus	0	1	Paratya_cf._typa	1	0
Palaemon_mercedae	1	0	Paratya_compressa	1	0
Palaemon_mesogenitor	1	0	Paratya_curvirostris	1	0
Palaemon_mesopotamicus	1	0	Paratya_howensis	1	0
Palaemon_modestus	0	1	Paratya_improvisa	1	0
Palaemon_mundusnovus	1	0	Paratya_norfolkensis	1	0
Palaemon_northropi	0	1	Parhippolyte_cf._uveae	0	1
Palaemon_octaviae	0	1	Parhippolyte_sterreri	0	1
Palaemon_orientis	0	1	Parisia_gracilis	1	0
Palaemon_ortmanni	0	1	Parisia_unguis	1	0
Palaemon_pacificus	0	1	Pasiphaea_planidorsalis	0	1
Palaemon_paludosus	1	0	Pasiphaea_sirenkoi	0	1
Palaemon_pandaliformis	1	0	Pasiphaea_sivado	0	1
Palaemon_paucidens	0	1	Pasiphaea_telacantha	0	1
Palaemon_peringueyi	0	1	Periclimenaeus_bidentatus	0	1
Palaemon_peruanus	0	1	Periclimenaeus_tuamotae	0	1
Palaemon_pugio	1	0	Periclimenella_spinifera	0	1
Palaemon_ritteri	0	1	Periclimenes_boucheti	0	1
Palaemon_schmitti	0	1	Periclimenes_brevicarpalis	0	1
Palaemon_semmelinkii	0	1	Periclimenes_commensalis	0	1
Palaemon_serenus	0	1	Periclimenes_dentidactylus	0	1
Palaemon_serratus	0	1	Periclimenes_digitalis	0	1
Palaemon_serrifer	0	1	Periclimenes_hertwigi	0	1
Palaemon_sinensis	0	1	Periclimenes_imperator	0	1
Palaemon_tonkinensis	1	0	Periclimenes_laccadivensis	0	1
Palaemon_turcorum	1	0	Periclimenes_leptunguis	0	1
Palaemon_varians	1	0	Periclimenes_ngi	0	1
Palaemon_vulgaris	1	0	Periclimenes_parvus	0	1
Palaemon_xiphias	0	1	Periclimenes_sandybrucei	0	1
Palaemon_zariquieyi	0	1	Periclimenes_soror	0	1
Palaemonella_holmesi	0	1	Peripandalus	0	1
Palaemonella_pottsi	0	1	Philarius_gerlachei	0	1
Palaemonella_rotumana	0	1	Philarius_imperialis	0	1
Palaemonias_sp.1	1	0	Philarius_minor	0	1
Palaemonias_sp.2	1	0	Philocheras	0	1
Pandalina	0	1	Phycomenes_cobourgi	0	1
Pandalopsis_dispar	0	1	Phyllognathia_ceratophthalma	0	1
Pandalopsis_lamelligera	0	1	Platycaris_latirostris	0	1
Pandalus_borealis	0	1	Plesionika_acanthonotus	0	1

Supplementary Table 2

Taxon	Freshwater/ anchialine	Marine	Taxon	Freshwater/ anchialine	Marine
Plesionika_antigai	0	1	Synalpheus_aff._apioceros_A	0	1
Plesionika_edwardsii	0	1	Synalpheus_aff._apioceros_B	0	1
Plesionika_heterocarpus	0	1	Synalpheus_aff._longicarpus	0	1
Plesionika_longipes	0	1	Synalpheus_aff._paraneptunus	0	1
Plesionika_martia	0	1	Synalpheus_africanus	0	1
Plesionika_scopifera	0	1	Synalpheus_agelas	0	1
Pliopontonia_furtiva	0	1	Synalpheus_anasimus	0	1
Pomagnathus_corallinus	0	1	Synalpheus_androsi	0	1
Pontocaris	0	1	Synalpheus_antillensis	0	1
Pontophilus_norvegicus	0	1	Synalpheus_apioceros	0	1
Potamalpheops_monodi	1	0	Synalpheus_arostris	0	1
Potamalpheops_pylorus	1	0	Synalpheus_belizensis	0	1
Potimirim_glabra	1	0	Synalpheus_bocas	0	1
Potimirim_mexicana	1	0	Synalpheus_brevicarpus	0	1
Potimirim_potimirim	1	0	Synalpheus_brevifrons	0	1
Potimirim_sp.	1	0	Synalpheus_brooksi	0	1
Prionalpheus_triarticulatus	0	1	Synalpheus_carpenteri	0	1
Prionocrangon	0	1	Synalpheus_cf._africanus_B	0	1
Procaris_ascensionis	1	0	Synalpheus_cf._brooksi	0	1
Procaris_mexicana	1	0	Synalpheus_cf._occidentalis	0	1
Processa_guyanae	0	1	Synalpheus_cf._paraneptunus	0	1
Procletes	0	1	Synalpheus_chacei	0	1
Psalidopus_huxleyi	0	1	Synalpheus_charon	0	1
Pseudathanas_darwiniensis	0	1	Synalpheus_corallinus	0	1
Pseudopalaemon_amazonensis	1	0	Synalpheus_dardeaui	0	1
Pseudopalaemon_bouvieri	1	0	Synalpheus_digueti	0	1
Pseudopalaemon_chryseus	1	0	Synalpheus_duffyi	0	1
Pseudopalaemon_gouldingi	1	0	Synalpheus_fritzmuelleri	0	1
Pterocaris_typica	0	1	Synalpheus_gambarelloides	0	1
Pycneus_morsitans	1	0	Synalpheus_goodei	0	1
Pycnisia_raptor	1	0	Synalpheus_gracilirostris	0	1
Racilius_compressus	0	1	Synalpheus_guerini	0	1
Rhynchocinetes_australis	0	1	Synalpheus_hemphilli	0	1
Rhynchocinetes_balssi	0	1	Synalpheus_hoetjesi	0	1
Rhynchocinetes_brucei	0	1	Synalpheus_idios	0	1
Rhynchocinetes_conspiciocellus	0	1	Synalpheus_kensleyi	0	1
Rhynchocinetes_durbanensis	0	1	Synalpheus_lani	0	1
Rhynchocinetes_typus	0	1	Synalpheus_longicarpus	0	1
Rhynchocinetes_uritai	0	1	Synalpheus_mcclendoni	0	1
Rhynocrangon	0	1	Synalpheus_mexicanus	0	1
Rimicaris_exoculata	0	1	Synalpheus_microneptunus	0	1
Rimicaris_hybisae	0	1	Synalpheus_minus	0	1
Rugathanas_borradailei	0	1	Synalpheus_near_sanctithomae	0	1
Sabinea	0	1	Synalpheus_nilandensis	0	1
Salmoneus_serratidigitus	0	1	Synalpheus_nobilii	0	1
Salmoneus_tricristatus	0	1	Synalpheus_obtusifrons	0	1
Saron_marmoratus	0	1	Synalpheus_paraneptunus	0	1
Saron_sp.	0	1	Synalpheus_peruvianus	0	1
Sclerocrangon	0	1	Synalpheus_plumosetosus	0	1
Sinodina_sp.	1	0	Synalpheus_ruetzleri	0	1
Stenalpheops_crangonus	0	1	Synalpheus_rufus	0	1
Stygiocaris_lancifera	1	0	Synalpheus_sanctithomae	0	1
Stygiocaris_sp.	1	0	Synalpheus_sanjosei	0	1
Stygiocaris_stylifera	1	0	Synalpheus_sanlucasi	0	1
Stylodactylus_libratus	0	1	Synalpheus_stylopleuron	0	1
Stylodactylus_major	0	1	Synalpheus_superus	0	1
Stylopandalus_richardi	0	1	Synalpheus_thele	0	1
Synalpheus_aff._antillensis	0	1	Synalpheus_townsendi	0	1

Supplementary Table 2

Taxon	Freshwater/ anchialine	Marine	Taxon	Freshwater/ anchialine	Marine
Synalpheus_ul	0	1	Yagerocaris_cozumel	1	0
Synalpheus_wickstenae	0	1			
Synalpheus_williamsi	0	1			
Synalpheus_yano	0	1			
Syncaris_pacifica	1	0			
Systellaspis_cristata	0	1			
Systellaspis_debilis	0	1			
Systellaspis_pellucida	0	1			
Thalassocaris_crinita	0	1			
Thaumastocaris_streptopus	0	1			
Thinora_maldivensis	0	1			
Thor_amboinensis	0	1			
Thor_cf._manningi	0	1			
Tozeuma_lanceolatum	0	1			
Tozeuma_sp.	0	1			
Trachycaris_rugosa	0	1			
Trachycaris_sp.	0	1			
Troglindicus_phreaticus	1	0			
Troglocaris_anophthalmus	1	0			
Troglocaris_bosnica	1	0			
Troglocaris_cf._fagei	1	0			
Troglocaris_cf._osterloffi	1	0			
Troglocaris_hercegovinensis	1	0			
Troglocaris_kapelana	1	0			
Troglocaris_neglecta	1	0			
Troglocaris_planinensis	1	0			
Troglocaris_prasence	1	0			
Troglocaris_pretneri	1	0			
Troglocaris_sp._Bosnian_Clade	1	0			
Troglocubanus_calcis	1	0			
Troglocubanus_eigenmanni	1	0			
Troglocubanus_gibarensis	1	0			
Troglocubanus_inermis	1	0			
Troglocubanus_jamaicensis	1	0			
Troglomexicanus_perezfarfanteae	1	0			
Tuleariocaris_zanzibarica	0	1			
Typhlatya_arfeae	1	0			
Typhlatya_consobrina	1	0			
Typhlatya_dzilamensis	1	0			
Typhlatya_garciai	1	0			
Typhlatya_iliffei	1	0			
Typhlatya_kakuki	1	0			
Typhlatya_miravetensis	1	0			
Typhlatya_mitchelli	1	0			
Typhlatya_monae	1	0			
Typhlatya_pearsei	1	0			
Typhlatya_rogersi	1	0			
Typhlatya_sp.	1	0			
Typhlatya_taina	1	0			
Typhlocaris_salentina	1	0			
Unguicaris_panglaonis	0	1			
Unguicaris_pilipes	0	1			
Urocaridella_antonbruunii	0	1			
Urocaridella_pulchella	0	1			
Urocaris_longicaudata	0	1			
Vercoia	0	1			
Vexillipar_repandum	0	1			
Xiphocaris_elongata	1	0			

Supplementary Table 3

Taxon	Symbiont	Non-Symbiont	Taxon	Symbiont	Non-Symbiont
Acanthephyra_acutifrons	0	1	Anchistioides_sp.1	1	0
Acanthephyra_cucullata	0	1	Anchistus_custoides	1	0
Acanthephyra_curtirostris	0	1	Anchistus_custos	1	0
Acanthephyra_media	0	1	Anchistus_demani	1	0
Acanthephyra_pelagica	0	1	Anchistus_miersi	1	0
Acanthephyra_purpurea	0	1	Ancylomenes_holthuisi	1	0
Acanthephyra_quadrispinosa	0	1	Ancylomenes_luteomaculatus	1	0
Agostocaris_sp.	0	1	Ancylomenes_venustus	1	0
Alope_orientalis	0	1	Anisomenaeus_spinimanus	1	0
Alpheopsis_aequalis	0	1	Antecaridina_lauensis	0	1
Alpheopsis_trispinosa	0	1	Antecaridina_sp.2	0	1
Alpheus_agilis	0	1	Arete_indicus	1	0
Alpheus_amblyonyx	0	1	Aretopsis_amabilis	1	
Alpheus_antepaenultimus	0	1	Argis	0	1
Alpheus_bouvieri	0	1	Athanas_dimorphus	1	0
Alpheus_chacei	0	1	Athanas_nitescens	0	1
Alpheus_colombiensis	0	1	Athanas_squillophilus	1	0
Alpheus_cristulifrons	0	1	Athanopsis_australis	1	0
Alpheus_cylindricus	0	1	Athanopsis_brevirostris	1	0
Alpheus_dentipes	0	1	Athanopsis_dentipes	1	0
Alpheus_edwardsii	0	1	Athanopsis_platyrhynchus	1	0
Alpheus_estuariensis	0	1	Athanopsis_rubricinctuta	1	0
Alpheus_floridanus	0	1	Atya_gabonensis	0	1
Alpheus_formosus	0	1	Atya_innocous	0	1
Alpheus_galapagensis	0	1	Atya_lanipes	0	1
Alpheus_hebes	0	1	Atya_margaritacea	0	1
Alpheus_javieri	0	1	Atya_ortmannioides	0	1
Alpheus_latus	0	1	Atya_scabra	0	1
Alpheus_macrocheles	0	1	Atyaephyra_orientalis	0	1
Alpheus_malleator	0	1	Atyaephyra_stankoi	0	1
Alpheus_normanni	0	1	Atyaephyra_strymonensis	0	1
Alpheus_nuttingi	0	1	Atyaephyra_thyamisensis	0	1
Alpheus_panamensis	0	1	Atyella_brevirostris	0	1
Alpheus_paracrinitus	0	1	Atyoida_bisulcata	0	1
Alpheus_peasei	0	1	Atyoida_pilipes	0	1
Alpheus_rapacida	0	1	Atyopsis_moluccensis	0	1
Alpheus_rostratus	0	1	Atyopsis_spinipes	0	1
Alpheus_saxidomus	0	1	Australatya_striolata	0	1
Alpheus_schmitti	0	1	Austropandalus	0	1
Alpheus_simus	0	1	Automate_dolichognatha	0	1
Alpheus_sulcatus	0	1	Automate_evermanni	0	1
Alpheus_tenuis	0	1	Automate_hayashii	0	1
Alpheus_thomasi	0	1	Bannereus_anomalus	1	0
Alpheus_umbo	0	1	Barbouria_cubensis	0	1
Alpheus_viridari	0	1	Batella_parvimanus	1	0
Alpheus_websteri	0	1	Bathyhippolyte_yaldwyni	0	1
Alvinocaridinides_formosa	0	1	Bathypalaemonella_serratipalma	1	0
Alvinocaris_longirostris	0	1	Bathypalaemonetes_brevirostris	1	0
Alvinocaris_lusca	0	1	Bermudacaris_harti	0	1
Alvinocaris_markensis	0	1	Brachycarpus_biunguiculatus	0	1
Alvinocaris_muricola	0	1	Brachycarpus_crosnieri	0	1
Alvinocaris_sp.1	0	1	Bythocaris_leucopis	0	1
Alvinocaris_sp.2	0	1	Calliasmata_nohochi	0	1
Alvinocaris_stactophila	0	1	Calliasmata_pholidota	0	1
Ambidexter_symmetricus	0	1	Campylonotus_capensis	0	1
Anachlorocurtis_commensalis	1	0	Caridella_paski	0	1
Anachlorocurtis_occidentalis	1	0	Caridina_africana	0	1
Anchiopontonia_hurii	1	0	Caridina_brachydactyla	0	1
Anchistioides_antiguensis	1	0	Caridina_cantonensis	0	1

Supplementary Table 3

Taxon	Symbiont	Non-Symbiont	Taxon	Symbiont	Non-Symbiont
Caridina_cf._acutirostris	0	1	Chlorotocoides	0	1
Caridina_cf._imitatrix	0	1	Chorismus_antarcticus	0	1
Caridina_cf._nilotica	0	1	Chorocaris_chacei	0	1
Caridina_cf._weberi	0	1	Cineotorhynchus_cf._rigens	0	1
Caridina_confusa	0	1	Cinetorhynchus_erythrostictus	0	1
Caridina_cruzi	0	1	Cinetorhynchus_hendersoni	0	1
Caridina_fernandoi	0	1	Cinetorhynchus_reticulatus	0	1
Caridina_gracilipes	0	1	Cinetorhynchus_striatus	0	1
Caridina_gracilirostris	0	1	Conchodytes_biunguiculatus	1	0
Caridina_lanceolata	0	1	Conchodytes_chadi	1	0
Caridina_longirostris	0	1	Conchodytes_meleagrinae	1	0
Caridina_multidentata	0	1	Conchodytes_monodactylus	1	0
Caridina_opaensis	0	1	Conchodytes_placunae	1	0
Caridina_pristis	0	1	Conchodytes_pteriae	1	0
Caridina_propinqua	0	1	Coralliocaris_graminea	1	0
Caridina_pseudodenticulata	0	1	Coralliocaris_superba	1	0
Caridina_sarasinorum	0	1	Coronalpheus_natator	0	1
Caridina_serrata	0	1	Crangon_franciscorum	0	1
Caridina_serratirostris	0	1	Crangon_septemspinosa	0	1
Caridina_sp.	0	1	Creaseria_morleyi	0	1
Caridina_sp.1	0	1	Cryphiops_brasiliensis	0	1
Caridina_sp.17	0	1	Cryphiops_caementarius	0	1
Caridina_sp.18	0	1	Cryphiops_luscus	0	1
Caridina_sp.19	0	1	Cuapetes_amymone	1	0
Caridina_sp.2	0	1	Cuapetes_anacanthus	0	1
Caridina_sp.2	0	1	Cuapetes_andamanensis	0	1
Caridina_sp.21	0	1	Cuapetes_elegans	0	1
Caridina_sp.22	0	1	Cuapetes_ensifrons	0	1
Caridina_sp.23	0	1	Cuapetes_grandis	0	1
Caridina_sp.3	0	1	Cuapetes_tenuipes	0	1
Caridina_sp.4	0	1	Dactylonia_ascidicola	1	0
Caridina_sp.5	0	1	Dactylonia_holthuisi	1	0
Caridina_sp.6	0	1	Dactylonia_monnioti	1	0
Caridina_sp.7	0	1	Dactylonia_okai	1	0
Caridina_sp.8	0	1	Deioneus_sandizelli	0	1
Caridina_sp.9	0	1	Dichelopandalus_bonnieri	0	1
Caridina_sp.A1	0	1	Discias_sp.	1	0
Caridina_sp.A2	0	1	Dorodotes	0	1
Caridina_sp.B1	0	1	Dugastella_marocana	0	1
Caridina_sp.B1	0	1	Dugastella_valentina	0	1
Caridina_sp.B4	0	1	Edoneus_marulas	0	1
Caridina_sp.B6	0	1	Ephyrina_bifida	0	1
Caridina_sp.B7	0	1	Ephyrina_figueirai	0	1
Caridina_sp.B9	0	1	Ephyrina_ombango	0	1
Caridina_sp.C	0	1	Eualus_barbatus	0	1
Caridina_sp.D	0	1	Eualus_biunguis	0	1
Caridina_spelunca	0	1	Eualus_cranchii	0	1
Caridina_steineri	0	1	Eualus_fabricii	0	1
Caridina_sumatrensis	0	1	Eualus_suckleyi	0	1
Caridina_thermophila	0	1	Eugonatonotus_chacei	0	1
Caridina_thomasi	0	1	Eugonatonotus_crassus	0	1
Caridina_togoensis	0	1	Exhippolysmata_ensirostris	0	1
Caridina_trifasciata	0	1	Exhippolysmata_oplophoroides	0	1
Caridina_typus	0	1	Gallocaris_inermis	0	1
Caridina_weberi	0	1	Gelastocaris_paronae	1	0
Caridina_zebra	0	1	Glyphocrangon_perplexa	0	1
Caridinides_wilkinsi	0	1	Glyphocrangon_stenolepis	0	1
Chlorocurtis	0	1	Gnathophylloides_mineri	1	0
Chlorotocella	0	1	Gnathophyllum_americanum	1	0

Supplementary Table 3

Taxon	Symbiont	Non-Symbiont	Taxon	Symbiont	Non-Symbiont
Halocaridina_rubra	0	1	Lysmata_ankeri	0	1
Halocaridinides_trigonophthalma	0	1	Lysmata_argentopunctata	0	1
Harpiliopsis_beaupresii	1	0	Lysmata_bahia	0	1
Harpiliopsis_spinigera	1	0	Lysmata_boggessi	0	1
Harpilius_lutescens	1	0	Lysmata_californica	0	1
Heptacarpus_futilirostris	0	1	Lysmata_cf._anchisteus	0	1
Heptacarpus_geniculatus	0	1	Lysmata_cf._intermedia	0	1
Heptacarpus_palpator	0	1	Lysmata_cf._ternatensis	0	1
Heterocarpus_amacula	0	1	Lysmata_debelius	0	1
Heterocarpus_calmani	0	1	Lysmata_galapagensis	0	1
Heterocarpus_dorsalis	0	1	Lysmata_grabhami	0	1
Heterocarpus_gibbosus	0	1	Lysmata_gracilirostris	0	1
Heterocarpus_hayashii	0	1	Lysmata_hochi	0	1
Heterocarpus_intermedius	0	1	Lysmata_holthuisi	0	1
Heterocarpus_laevigatus	0	1	Lysmata_intermedia	0	1
Heterocarpus_parvispina	0	1	Lysmata_moorei	0	1
Heterocarpus_sibogae	0	1	Lysmata_nayaritensis	0	1
Heterocarpus_woodmasoni	0	1	Lysmata_new_species_cf._vittata	0	1
Heterogenys_microphthalma	0	1	Lysmata_nilita	0	1
Hippolyte_obliquimanus	0	1	Lysmata_olavoi	0	1
Hippolyte_pleuracantha	0	1	Lysmata_pederseni	1	0
Hippolyte_varians	0	1	Lysmata_rafa	0	1
Hymenocera_picta	0	1	Lysmata_seticaudata	0	1
Hymenodora_glacialis	0	1	Lysmata_udoi	0	1
Hymenodora_gracilis	0	1	Lysmata_vittata	0	1
Ischnopontonia_lophos	1	0	Lysmata_wurdemanni	0	1
Izucaris_masudai	1	0	Lysmatella_prima	0	1
Janicea_antiguensis	0	1	Macrobrachium_abrahami	0	1
Janicella_spinicauda	0	1	Macrobrachium_acanthurus	0	1
Jocaste_lucina	1	0	Macrobrachium_aemulum	0	1
Jonga_serrei	0	1	Macrobrachium_amazonicum	0	1
Lancaris_kumariae	0	1	Macrobrachium_americanum	0	1
Lancaris_singhalensis	0	1	Macrobrachium_aracamuni	0	1
Laomenes_amboinensis	1	0	Macrobrachium_asperulum	0	1
Laomenes_ceratophthalmus	1	0	Macrobrachium_australe	0	1
Laomenes_pardus	1	0	Macrobrachium_birai	0	1
Latreutes_fucorum	0	1	Macrobrachium_borellii	0	1
Latreutes_pymoeus	0	1	Macrobrachium_brasiliense	0	1
Leander_sp.	0	1	Macrobrachium_carcinus	0	1
Leander_tenuicornis	0	1	Macrobrachium_cf_nipponense	0	1
Lebbeus_antarcticus	0	1	Macrobrachium_cf_pilimanus	0	1
Lebbeus_groenlandicus	0	1	Macrobrachium_cf._horstii	0	1
Lebbeus_laurentae	0	1	Macrobrachium_cortezi	0	1
Lebbeus_polaris	0	1	Macrobrachium_crenulatum	0	1
Lebbeus_virentova	0	1	Macrobrachium_dacqueti	0	1
Leptocarpus_potamiscus	0	1	Macrobrachium_depressimanum	0	1
Leptochela_bermudensis	0	1	Macrobrachium_digueti	0	1
Leptochela_carinata	0	1	Macrobrachium_dux	0	1
Leptochela_papulata	0	1	Macrobrachium_edentatum	0	1
Leptopalaemon_glabrus	0	1	Macrobrachium_esculentum	0	1
Leptopalaemon_sp.1	0	1	Macrobrachium_ferreirai	0	1
Leptopalaemon_sp.2	0	1	Macrobrachium_foai	0	1
Leptopalaemon_sp.3	0	1	Macrobrachium_formosense	0	1
Limnocaridina_parvula	0	1	Macrobrachium_fukienense	0	1
Limnocaridina_similis	0	1	Macrobrachium_gracilirostre	0	1
Limnocaridina_tanganyikae	0	1	Macrobrachium_grandimanus	0	1
Lipkemenes_lanipes	1	0	Macrobrachium_hainanense	0	1
Lissosabinea	0	1	Macrobrachium_hancocki	0	1
Lysmata_amboinensis	0	1	Macrobrachium_heterochirus	0	1

Supplementary Table 3

Taxon	Symbiont	Non-Symbiont	Taxon	Symbiont	Non-Symbiont
Macrobrachium_holthuisi	0	1	Macrobrachium_trompii	0	1
Macrobrachium_iheringi	0	1	Macrobrachium_venustum	0	1
Macrobrachium_inflatum	0	1	Macrobrachium_villalobosi	0	1
Macrobrachium_inpa	0	1	Macrobrachium_yui	0	1
Macrobrachium_jaroense	0	1	Macrobrachium_zariquieyi	0	1
Macrobrachium_lamarrei	0	1	Manipontonia_psamathe	1	0
Macrobrachium_lanatum	0	1	Marosina_longirostris	0	1
Macrobrachium_lar	0	1	Meningodora_mollis	0	1
Macrobrachium_latidactylus	0	1	Meningodora_sp.	0	1
Macrobrachium_latimanus	0	1	Meningodora_vesca	0	1
Macrobrachium_lepidactyloides	0	1	Merguia_oligodon	0	1
Macrobrachium_lujae	0	1	Merguia_rhizophorae	0	1
Macrobrachium_maculatum	0	1	Metabetaeus_minutus	0	1
Macrobrachium_malayanum	0	1	Metabetaeus_sp.	0	1
Macrobrachium_malcolmsonii	0	1	Metacrangon_sp.	0	1
Macrobrachium_michoacanus	0	1	Metalpheus_rostratipes	0	1
Macrobrachium_naso	0	1	Micratya_poeyi	0	1
Macrobrachium_nattereri	0	1	Micratya_sp.	0	1
Macrobrachium_neglectum	0	1	Mimocaris_heterocarpoides	0	1
Macrobrachium_nepalense	0	1	Mirocaris_fortunata	0	1
Macrobrachium_niphanae	0	1	Miropandalus_hardingi	1	0
Macrobrachium_nipponense	0	1	Nematocarcinus_cursor	0	1
Macrobrachium_novaehollandiae	0	1	Nematocarcinus_rotundus	0	1
Macrobrachium_occidentale	0	1	Nematopalaemon_schmitti	0	1
Macrobrachium_ohione	0	1	Nennalpheus_inarticulatus	0	1
Macrobrachium_panamense	0	1	Nennalpheus_sibogae	0	1
Macrobrachium_pectinatum	0	1	Neocaridina_denticulata	0	1
Macrobrachium_petronioi	0	1	Neocaridina_palmata	0	1
Macrobrachium_pilimanus	0	1	Neocaridina_saccam	0	1
Macrobrachium_placidulum	0	1	Neocrangon_sagamiensis	0	1
Macrobrachium_placidum	0	1	Neopalaemon_nahuatlus	0	1
Macrobrachium_platycheles	0	1	Neostylodactylus_amarynthis	0	1
Macrobrachium_potiuna	0	1	Nikoides_schmitti	0	1
Macrobrachium_prabhakarani	0	1	Nippontonia_christellae	1	0
Macrobrachium_pumilum	0	1	Notocrangon	0	1
Macrobrachium_qilianensis	0	1	Notopandalus	0	1
Macrobrachium_quelchi	0	1	Notostomus_elegans	0	1
Macrobrachium_raridens	0	1	Notostomus_gibbosus	0	1
Macrobrachium_reyesi	0	1	Ogyrides_orientalis	0	1
Macrobrachium_rodriguezi	0	1	Ogyrides_sp.	0	1
Macrobrachium_rosenbergii	0	1	Onycocaris_quadratophthalma	1	0
Macrobrachium_rude	0	1	Oplophorus_gracilirostris	0	1
Macrobrachium_sankolli	0	1	Oplophorus_typus	0	1
Macrobrachium_scabriculum	0	1	Orthopontonia_ornata	1	0
Macrobrachium_shokitai	0	1	Palaemon_adspersus	0	1
Macrobrachium_sp._nov._1	0	1	Palaemon_africanus	0	1
Macrobrachium_sp._nov._2	0	1	Palaemon_annandalei	0	1
Macrobrachium_sp.1	0	1	Palaemon_antennarius	0	1
Macrobrachium_sp.2	0	1	Palaemon_antrorum	0	1
Macrobrachium_sp.3	0	1	Palaemon_argentinus	0	1
Macrobrachium_sp.4	0	1	Palaemon_atrinubes	0	1
Macrobrachium_sp.5	0	1	Palaemon_carinicauda	0	1
Macrobrachium_sp.6	0	1	Palaemon_carteri	0	1
Macrobrachium_sp.7	0	1	Palaemon_concinus	0	1
Macrobrachium_sp.8	0	1	Palaemon_cummingi	0	1
Macrobrachium_sp.nov._GUI	0	1	Palaemon_debilis	0	1
Macrobrachium_surinamicum	0	1	Palaemon_elegans	0	1
Macrobrachium_tenellum	0	1	Palaemon_floridanus	0	1
Macrobrachium_tolmerum	0	1	Palaemon_gladiator	0	1

Supplementary Table 3

Taxon	Symbiont	Non-Symbiont	Taxon	Symbiont	Non-Symbiont
Palaemon_gracilis	0	1	Parabetaeus_culliereti	0	1
Palaemon_gravieri	0	1	Parabetaeus_euryone	0	1
Palaemon_guangdongensis	0	1	Paracrangon	0	1
Palaemon_hancocki	0	1	Parapontocaris_aspera	0	1
Palaemon_hiltoni	0	1	Parapontocaris_levigata	0	1
Palaemon_holthuisi	0	1	Parapontophilus_gracilis	0	1
Palaemon_intermedius	0	1	Parapontophilus_junceus	0	1
Palaemon_ivonicus	0	1	Paratya_australiensis	0	1
Palaemon_litoreus	0	1	Paratya_cf._caledonica	0	1
Palaemon_longirostris	0	1	Paratya_cf._intermedia	0	1
Palaemon_macrodactylus	0	1	Paratya_cf._typa	0	1
Palaemon_maculatus	0	1	Paratya_compressa	0	1
Palaemon_mercedae	0	1	Paratya_curvirostris	0	1
Palaemon_mesogenitor	0	1	Paratya_howensis	0	1
Palaemon_mesopotamicus	0	1	Paratya_improvisa	0	1
Palaemon_modestus	0	1	Paratya_norfolkensis	0	1
Palaemon_mundusnovus	0	1	Parhippolyte_cf._uveae	0	1
Palaemon_northropi	0	1	Parhippolyte_sterreri	0	1
Palaemon_octaviae	0	1	Parisia_gracilis	0	1
Palaemon_orientis	0	1	Parisia_unguis	0	1
Palaemon_ortmanni	0	1	Pasiphaea_planidorsalis	0	1
Palaemon_pacificus	0	1	Pasiphaea_sirenkoi	0	1
Palaemon_paludosus	0	1	Pasiphaea_sivado	0	1
Palaemon_pandaliformis	0	1	Pasiphaea_telacantha	0	1
Palaemon_paucidens	0	1	Periclimenaeus_bidentatus	1	0
Palaemon_peringueyi	0	1	Periclimenaeus_tuamotae	1	0
Palaemon_peruanus	0	1	Periclimenella_spinifera	1	0
Palaemon_pugio	0	1	Periclimenes_boucheti	1	0
Palaemon_ritteri	0	1	Periclimenes_brevicarpalis	1	0
Palaemon_schmitti	0	1	Periclimenes_Symbiontis	1	0
Palaemon_semmelinkii	0	1	Periclimenes_dentidactylus	1	0
Palaemon_serenus	0	1	Periclimenes_digitalis	1	0
Palaemon_serratus	0	1	Periclimenes_hertwigi	1	0
Palaemon_serrifer	0	1	Periclimenes_imperator	1	0
Palaemon_sinensis	0	1	Periclimenes_laccadivensis	1	0
Palaemon_tonkinensis	0	1	Periclimenes_leptunguis	1	0
Palaemon_turcorum	0	1	Periclimenes_ngi	1	0
Palaemon_varians	0	1	Periclimenes_parvus	1	0
Palaemon_vulgaris	0	1	Periclimenes_sandybrucei	1	0
Palaemon_xiphias	0	1	Periclimenes_soror	1	0
Palaemon_zariquieyi	0	1	Peripandalus	0	1
Palaemonella_holmesi	0	1	Philarius_gerlachei	1	0
Palaemonella_pottsi	1	0	Philarius_imperialis	1	0
Palaemonella_rotumana	1	0	Philarius_minor	1	0
Palaemonias_sp.1	0	1	Philocheras	0	1
Palaemonias_sp.2	0	1	Phycomenes_cobourgi	0	1
Pandalina	0	1	Phyllognathia_ceratophthalma	1	0
Pandalopsis_dispar	0	1	Platycaris_latirostris	1	0
Pandalopsis_lamelligera	0	1	Plesionika_acanthonotus	0	1
Pandalus_borealis	0	1	Plesionika_antigai	0	1
Pandalus_danae	0	1	Plesionika_edwardsii	0	1
Pandalus_goniurus	0	1	Plesionika_heterocarpus	0	1
Pandalus_hypsinotus	0	1	Plesionika_longipes	0	1
Pandalus_jordani	0	1	Plesionika_martia	0	1
Pandalus_latirostris	0	1	Plesionika_scopifera	0	1
Pandalus_montagui	0	1	Pliopontonia_furtiva	1	0
Pandalus_prensor	0	1	Pomagnathus_corallinus	0	1
Pandalus_stenolepis	0	1	Pontocaris	0	1
Pantomus	0	1	Pontophilus_norvegicus	0	1

Supplementary Table 3

Taxon	Symbiont	Non-Symbiont	Taxon	Symbiont	Non-Symbiont
Potamalpheops_monodi	0	1	Synalpheus_belizensis	1	0
Potamalpheops_pylorus	0	1	Synalpheus_bocas	1	0
Potimirim_glabra	0	1	Synalpheus_brevicarpus	0	1
Potimirim_mexicana	0	1	Synalpheus_brevifrons	1	0
Potimirim_potimirim	0	1	Synalpheus_brooksi	1	0
Potimirim_sp.	0	1	Synalpheus_carpenteri	1	0
Prionalpheus_triarticulatus	0	1	Synalpheus_cf._africanus_B	0	1
Prionocrangon	0	1	Synalpheus_cf._brooksi	1	0
Procaris_ascensionis	0	1	Synalpheus_cf._occidentalis	0	1
Procaris_mexicana	0	1	Synalpheus_cf._paraneptunus	0	1
Processa_guyanae	0	1	Synalpheus_chacei	1	0
Procletes	0	1	Synalpheus_charon	1	0
Psalidopus_huxleyi	0	1	Synalpheus_corallinus	0	1
Pseudathanas_darwiniensis	0	1	Synalpheus_dardeaui	1	0
Pseudopalaemon_amazonensis	0	1	Synalpheus_digueti	0	1
Pseudopalaemon_bouvieri	0	1	Synalpheus_duffyi	1	0
Pseudopalaemon_chryseus	0	1	Synalpheus_fritzmuelleri	0	1
Pseudopalaemon_gouldingi	0	1	Synalpheus_gambarelloides	1	0
Pterocaris_typica	0	1	Synalpheus_goodei	1	0
Pycneus_morsitans	0	1	Synalpheus_gracilirostris	0	1
Pycnisia_raptor	0	1	Synalpheus_guerini	0	1
Racilius_compressus	1	0	Synalpheus_hemphilli	0	1
Rhynchocinetes_australis	0	1	Synalpheus_hoetjesi	1	0
Rhynchocinetes_balssi	0	1	Synalpheus_idios	1	0
Rhynchocinetes_brucei	0	1	Synalpheus_kensleyi	1	0
Rhynchocinetes_conspiciocellus	0	1	Synalpheus_lani	1	0
Rhynchocinetes_durbanensis	0	1	Synalpheus_longicarpus	0	1
Rhynchocinetes_typus	0	1	Synalpheus_mcclendoni	0	1
Rhynchocinetes_uritai	0	1	Synalpheus_mexicanus	0	1
Rhynocrangon	0	1	Synalpheus_microneptunus	1	0
Rimicaris_exoculata	0	1	Synalpheus_minus	0	1
Rimicaris_hybisae	0	1	Synalpheus_near_sanctithomae	0	1
Rugathanas_borradailei	0	1	Synalpheus_nilandensis	0	1
Sabinea	0	1	Synalpheus_nobilii	0	1
Salmoneus_serratidigitus	0	1	Synalpheus_obtusifrons	0	1
Salmoneus_tricristatus	0	1	Synalpheus_paraneptunus	1	0
Saron_marmoratus	0	1	Synalpheus_peruvianus	0	1
Saron_sp.	0	1	Synalpheus_plumosetosus	1	0
Sclerocrangon	0	1	Synalpheus_ruetzleri	1	0
Sinodina_sp.	0	1	Synalpheus_rufus	1	0
Stenalpheops_crangonus	1	0	Synalpheus_sanctithomae	1	0
Stygiocaris_lancifera	0	1	Synalpheus_sanjosei	0	1
Stygiocaris_sp.	0	1	Synalpheus_sanlucasi	0	1
Stygiocaris_stylifera	0	1	Synalpheus_stylopleuron	0	1
Stylodactylus_libratus	0	1	Synalpheus_superus	0	1
Stylodactylus_major	0	1	Synalpheus_thele	1	0
Stylopandalus_richardi	0	1	Synalpheus_townsendi	0	1
Synalpheus_aff._antillensis	0	1	Synalpheus_ul	1	0
Synalpheus_aff._apioceros_A	0	1	Synalpheus_wickstenae	0	1
Synalpheus_aff._apioceros_B	0	1	Synalpheus_williamsi	1	0
Synalpheus_aff._longicarpus	0	1	Synalpheus_yano	0	1
Synalpheus_aff._paraneptunus	0	1	Syncaris_pacifica	0	1
Synalpheus_africanus	0	1	Systellaspis_cristata	0	1
Synalpheus_agelas	1	0	Systellaspis_debilis	0	1
Synalpheus_anasimus	0	1	Systellaspis_pellucida	0	1
Synalpheus_androsi	1	0	Thalassocaris_crinita	0	1
Synalpheus_antillensis	0	1	Thaumastocaris_streptopus	1	0
Synalpheus_apioceros	0	1	Thinora_maldivensis	0	1
Synalpheus_arostris	0	1	Thor_amboinensis	1	0

Supplementary Table 3

Taxon	Symbiont	Non-Symbiont
Thor_cf._manningi	0	1
Tozeuma_lanceolatum	1	0
Tozeuma_sp.	0	1
Trachycaris_rugosa	0	1
Trachycaris_sp.	0	1
Troglindicus_phreaticus	0	1
Troglocaris_anophthalmus	0	1
Troglocaris_bosnica	0	1
Troglocaris_cf._fagei	0	1
Troglocaris_cf._osterloffi	0	1
Troglocaris_hercegovinensis	0	1
Troglocaris_kapelana	0	1
Troglocaris_neglecta	0	1
Troglocaris_planinensis	0	1
Troglocaris_prasence	0	1
Troglocaris_pretneri	0	1
Troglocaris_sp._Bosnian_Clade	0	1
Troglocubanus_calcis	0	1
Troglocubanus_eigenmanni	0	1
Troglocubanus_gibarensis	0	1
Troglocubanus_inermis	0	1
Troglocubanus_jamaicensis	0	1
Troglomexicanus_perezfarfanteae	0	1
Tuleariocaris_zanzibarica	1	0
Typhlatya_arfeae	0	1
Typhlatya_consobrina	0	1
Typhlatya_dzilamensis	0	1
Typhlatya_garciai	0	1
Typhlatya_iliffei	0	1
Typhlatya_kakuki	0	1
Typhlatya_miravetensis	0	1
Typhlatya_mitchelli	0	1
Typhlatya_monae	0	1
Typhlatya_pearsei	0	1
Typhlatya_rogersi	0	1
Typhlatya_sp.	0	1
Typhlatya_taina	0	1
Typhlocaris_salentina	0	1
Unguicaris_panglaonis	1	0
Unguicaris_pilipes	1	0
Urocaridella_antonbruunii	0	1
Urocaridella_pulchella	0	1
Urocaris_longicaudata	0	1
Vercoia	0	1
Vexillipar_repandum	0	1
Xiphocaris_elongata	0	1
Yagerocaris_cozumel	0	1

Supplementary references

[1] Revell, L. J. phytools: An R package for phylogenetic comparative biology (and other things). Methods Ecol. Evol. 3, 217-223 (2012).

[2] Rabosky, D. L. Automatic detection of key innovations, rate shifts, and diversity-dependence on phylogenetic trees. *PLoS One* **9,** e89543 (2014).

[3] Rabosky, D. L., Grundler, M., Anderson, C., Title, P., Shi, J. J., Brown, J. W., Huang, H. and Larson, J. G. BAMMtools: an R package for the analysis of evolutionary dynamics on phylogenetic trees. Methods Ecol Evol, 5: 701–707. doi:10.1111/2041-210X.12199 (2014).
