# Supplementary material for: Freshwater transitions and symbioses shaped the evolution and extant diversity of caridean shrimps
Source: Commun Biol. 2018 Feb 22;1:16. doi: 10.1038/s42003-018-0018-6 (PMC6123698; doi:10.1038/s42003-018-0018-6)
Supplement: Supplementary file 2 — Description of Additional Supplementary Files [file 42003_2018_18_MOESM2_ESM.docx]

**Description of Additional Supplementary Files**

File Name: Supplementary Data 1 – (Final source trees)

Description: Final source trees used in the analysis.

File Name: Supplementary Data 2 – (Source references)

Description: Bib file containing references for all source trees.

File Name: Supplementary Data 3 – (Rogue taxa)

Description: List of rogue taxa found by the analysis.

File Name: Supplementary Data 4 – (Time-calibrated supertree)

Description: Supertree in Nexus format.

File Name: Supplementary Data 5 - (Ancestral State Reconstruction code)

Description: R script used to calculate Ancestral State Reconstructions.

File Name: Supplementary Data 6 – (BAMM settings)

Description: Control file used for BAMM analysis.

File Name: Supplementary Data 7 – (Caridea taxon sampling)

Description: Species sampling file used during BAMM analysis.
